# Chinese university students’ preferences for physical activity incentive programs: a discrete choice experiment

**DOI:** 10.3389/fpubh.2023.1281740

**Published:** 2023-11-01

**Authors:** Jingbo Zhang, Qing Li, Jinzi Zhang, Xianqi Zhao, Maomin Jiang, Xincheng Huang, Diyue Liu, Yupei Yan, Xialei Li, Jiangyun Chen, Zheng Feei Ma, Xiyue Zhang, Wai-Kit Ming, Tak-hap Wong, Guanyun Yan, Yibo Wu

**Affiliations:** ^1^School of Humanities and Social Sciences, Harbin Medical University, Harbin, China; ^2^School of Social Development and Public Policy, Beijing Normal University, Beijing, China; ^3^School of Public Health, Shandong University, Jinan, China; ^4^School of Public Affairs, Xiamen University, Xiamen, China; ^5^School of Economics and Management, Beijing Institute of Graphic Communication, Beijing, China; ^6^International School of Public Health and One Health, Hainan Medical University, Haikou, China; ^7^Department of Humanities, Arts and Media, Changzhi Medical College, Changzhi, China; ^8^Department of Pharmacy Administration and Clinical Pharmacy, School of Pharmaceutical Sciences, Peking University, Beijing, China; ^9^School of Health Management, Southern Medical University, Guangzhou, China; ^10^Centre for Public Health and Wellbeing, School of Health and Social Wellbeing, College of Health, Science and Society, University of the West of England, Bristol, United Kingdom; ^11^Alliance Manchester Business School, University of Manchester, Manchester, United Kingdom; ^12^Department of Infectious Diseases and Public Health, Jockey Club College of Veterinary Medicine and Life Sciences, City University of Hong Kong, Hong Kong SAR, China; ^13^School of Public Health, Peking University, Beijing, China

**Keywords:** health behavior, management and policy, health promotion, willingness to accept, physical activity

## Abstract

**Purpose:**

This study aims to explore and compare Chinese university students’ preferences for various physical activity motivation programs.

**Patients and methods:**

A cross-sectional study was conducted in China from February 25 to March 25, 2022. Participants anonymously completed an online questionnaire based on a DCE. A total of 1,358 university students participated in the survey. The conditional logit model (CLM), willingness to accept (WTA), and propensity score matching (PSM) were used to assess college students’ preferences for different attributes and levels of physical activity incentive programs.

**Results:**

Respondents identified the number of bonus, exercise time, and academic rewards as the three most significant attributes of the athletic incentive program. The importance of each attribute varied based on individual characteristics such as gender and BMI. In CLM, college students displayed a preference for a “¥4” bonus amount (OR: 2.04, 95% CI 1.95–2.13), “20 min” of exercise time (OR: 1.85, 95% CI 1.79–1.92), and “bonus points for comprehensive test scores” as academic rewards (OR: 1.33, 95% CI 1.28–1.37). According to the WTA results, college students were willing to accept the highest cost to obtain academic rewards tied to composite test scores.

**Conclusion:**

The number of bonus, exercise time, and academic rewards emerge as the three most crucial attributes of physical activity incentive programs. Furthermore, college students with different characteristics exhibit heterogeneity in their preferences for such programs. These findings can guide the development of programs and policies aimed at motivating college students to engage in physical activities.

## 1. Introduction

Regular physical activity provides numerous health benefits, such as enhancing immunity, preventing non-communicable diseases, and enhancing mental well-being. For optimal health benefits, adults should avoid sedentary behavior and engage in a weekly routine of no less than 150–300 min of moderate-intensity aerobic physical exertion or at least 75–150 min of vigorous-intensity aerobic activity or a balanced combination of both types (1, 2). However, despite these benefits, a lack of physical activity remains a widespread global issue.

In a 2018 Lancet publication, the authors collected data from 358 research endeavors spanning 168 countries, revealing that 27.5% of 1.9 million individuals engaged in inadequate physical exercise (3). Physical inactivity and sedentary lifestyles are associated with conditions such as obesity, cancer, cardiovascular disease, and diabetes (4, 5). Due to the substantial time college students dedicate to classrooms and scholastic pursuits, they are particularly vulnerable to physical inactivity and sedentariness (6–8).

According to the SHoT study (Students’ Health and Well-being Study), the vast majority of college students’ physical activity levels fail to meet recommended standards. Meanwhile, the trend toward overweight and obesity among colleges students continues to surge (9, 10). As per the guidelines set by the People’s Republic of China’s Ministry of Education, students in traditional Chinese institutions are expected to meet certain physical education requirements. Specifically, students are expected to complete 144 h of physical education coursework within 4 years (11). Nonetheless, the issue of low physical exercise levels among Chinese university students remains an acute concern. Furthermore, insufficient physical activity among university students may exert a deleterious impact on physical literacy, amplify feelings of anxiety and despondency, and curtail the overall health-related quality of life (12–14).

In light of this, it is essential to devise appropriate policies or interventions to ameliorate college students’ physical activity levels. Given the substantial time spent in academia, schools play an indispensable role in promoting physical exercise among college attendees. Research shows a strong link between student physical activity and school policies (15). Moreover, studies have indicated the potential link between academic performance and the motivation to engage in physical activities. Schools can leverage academic achievements as incentives to encourage students to partake in physical endeavors (16); additionally, economic rewards emerge as a potent tool to motivate individuals to engage in physical activities (17).

Identifying individuals’ preferences concerning the type and timing of physical activity programs holds paramount importance (18, 19). In response to these preferences, policymakers could devise incentive programs that cater to college students’ reward preferences for physical exercise. Conducting a discrete choice experiment can help us understand college students’ preferences for physical activity incentive programs. Discrete Choice Experiments (DCE) stand as a prominent quantitative method in health economics and policy research. Built on the foundation of random utility theory, DCE enables the assessment of both individual and group preferences for various behaviors (20). This methodology has previously proven effective in estimating preferences related to physical activity within specific populations. For instance, it has been utilized to gauge preferences for physical activity among patients experiencing non-specific low back pain and preferences regarding financial incentives to encourage physical activity among older adults (21, 22).

However, despite the proven value of DCE in designing incentive programs to promote physical activity, no prior studies have specifically examined the preferences of Chinese college students for such programs. Recognizing the significance of college students as a demographic group whose physical activity habits profoundly impact their immediate health and long-term well-being, this study employed DCE. Our aim was to explore Chinese college students’ preferences and willingness to accept (WTA) incentive programs for physical activity. This exploration provides valuable insights into college students’ choices and preferences for these programs, contributing to the development of more effective health policies and interventions aimed at increasing physical activity levels among them.

## 2. Materials and methods

### 2.1. Discrete choice experiment

The foundation of Discrete Choice Experiment (DCE) lies in the random utility theory in economics. This method proposes that entities can be defined by a set of important attributes and their corresponding levels (e.g., test procedure, detection rate, test cost). Consequently, individuals mentally compare these qualities and levels in hypothetical scenarios before making choices among different options.

### 2.2. Identification of attributes and levels

Various methodologies are employed in ascertaining attributes and levels for DCE, including literature reviews, expert consultations, existing health outcome metrics, surveys, interviews, and focus groups. Esteemed scholars in the field advocate for the prioritization of qualitative approaches in identifying these attributes and levels (23). Such qualitative methods allow researchers to capture respondents’ perspectives, thus reducing the potential for attribute and level misspecification due to over-reliance on the researcher’s viewpoints (24, 25).

In this investigation, a comprehensive array of attributes and levels for motivational strategies in physical activity was compiled by drawing insights from pertinent literature and contextualizing them within the milieu of Chinese universities (26–30). An expert panel comprising two sports experts, two medical professionals, and two methodologists was convened to evaluate and appraise these attributes and levels. The panel received via email the attribute list, along with a concise overview. The experts individually assessed the attributes’ relevance, feasibility, and degrees, offering their invaluable insights. The researchers duly considered these valuable suggestions and compiled a refined list of attribute levels, which was subsequently presented to a focus group for further deliberation.

The focus group comprised five physical education teachers and five undergraduate university students, who engaged in detailed interviews about the questionnaire. They deemed the questionnaire to be thoughtfully prepared but requested clarification on some listed attributes and levels. Respondents pointed out minor spelling errors, which were promptly rectified. Importantly, the number of attribute levels and the questionnaire’s length were both deemed acceptable during the pretest. The completion of the survey took participants approximately 15–20 min. Aside from the minor spelling issues, the attributes and levels remained unchanged. Table 1 presents the six conclusively determined attributes concerning incentive techniques for physical exercise and their corresponding levels.

**Table 1:** Attributes and levels of physical activity incentive programs.

Attributes	Levels of attributes	Explain
Amount of bonus	¥1	The amount of prize money is set up to incentive university students to participate in the exercise incentive program, which is awarded at regular intervals.
	¥2	
	¥3	
	¥4	
Frequency of bonus payments	Paid every 4 weeks	Frequency of awarding prizes to university students participating in the exercise incentive program.
	Paid every 3 weeks	
	Paid every 2 weeks	
	Paid every 1 week	
Academic awards	Bonus points for moral education credits	Academic incentives for university students participating in the exercise incentive program.
	Bonus points for physical education test scores	
	Bonus points for comprehensive test scores	
Frequency of exercise	5 times a week	Frequency of training in an exercise incentive program.
	3 times a week	
	1 time a week	
Exercise time	60 min each time	Minimum time to be achieved in each exercise.
	40 min each time	
	20 min each time	
Conditions for receiving the award	Pass the physical fitness test	Conditions for participants to receive prizes and academic awards.
	Complete the exercise program on a regular basis and upload it to the online platform	
	Register for the exercise incentive program	

### 2.3. Experimental design and development of the questionnaire

Participants are invited to deliberate between diverse gradations of attributes, electing their preferred exercise incentive program. The DCE selection array is curated with six attributes, each featuring 3–4 tiers. An all-encompassing analytical design would entail 1,296 (4^2 × 3^4) potential choices, but this vastness proves excessive for a single survey and laborious for respondents to undertake. In pursuit of proportional inclusion of levels (level balancing) and to eliminate correlations among levels of distinct attributes, we devised a 16-choice set, employing a fractional ordinal orthogonal main effects design from the design compendium. Subsequently, respondents were randomly assigned to an 8-choice subset (orthogonality). To safeguard against any inherent bias in parameter estimation, we fashioned unlabeled choice experiments comprising three distinct choice scenarios, each harboring two discrete scenarios and an exit option.

The following sample size calculation formulas are commonly used in DCE studies. In the formula below, N stands for the minimum sample size advised, t for the number of tasks chosen, a for the number of choices made for each task, and c for the maximum number of attributes (31).


N≥500c/ta

Based on this formula, we determine *n* ≥ 125 (t = 8, a = 2, and c = 4). We intend to collect a sample of 1,250. There is a large number of samples, which ensures that the calculation will be accurate and reliable.

The questionnaire is divided into three distinct sections. The initial segment delves into respondents’ particulars, encompassing gender, age, household dynamics, academic level, scholastic achievements, living costs, BMI, and whether their parents are affiliated with the sports industry. Furthermore, we inquired about respondents’ visual health, mobile phone usage patterns, and habits related to smoking and alcohol consumption.

The second section of the survey employed the International Physical Activity Questionnaire Short Form (IPAQ-SF), a robust and reliable instrument comprising seven items, to gauge the participants’ physical health and activity levels (32). This comprehensive questionnaire appraises and computes three distinct intensities of activity: low-intensity activity (3.3 METs)(Metabolic Equivalent of Task, MET), moderate-intensity activity (4.0 METs), and high-intensity activity (8.0 METs). Respondents were requested to disclose the frequency and duration of their engagement in each intensity of activity, provided it persisted for at least 10 min (33). Based on the following formula, each participant’s total weekly exercise was calculated:


$$\begin{array}{l} \mathrm{Total}\;\mathrm{MET}-\mathrm{minutes}/\mathrm{week}=\mathrm{Low}\;PA\;\left(\mathrm{METs}\times \min\times \mathrm{days}\right)\\ \;+\;\mathrm{Moderate}\;PA\;\left(\mathrm{METs}\times \min\times \mathrm{days}\right)\\ \;+\;\mathrm{Vigorous}\;PA\;\left(\mathrm{METs}\times \min\times \mathrm{days}\right)\text{.} \end{array}$$

Based on the derived computations, the respondents were classified into three distinct tiers of physical activity: the low-activity group (<600 MET-minute/week), the moderate-activity group (≥600 MET-minute/week), and the high-activity group (≥3,000 MET-minute/week).

The third segment of the questionnaire probed the respondents to contemplate their favored physical activity incentive program within a thought-provoking three-task choice scenario. Each scenario required the respondents to envision themselves embarking on a physical activity incentive program comprising six attributes, each with a maximum of four levels. Nine task selection scenarios were presented to each respondent, with three alternatives offered for each scenario. The initial among the nine choice sets was designated as a fixed choice set. By including extreme options in this set, the validity of the DCE was rigorously ascertained. An example of a task selection scenario is depicted in Table 2.

**Table 2:** Example discrete choice experiment question.

	Option A	Option B	Option C
Amount of bonus	¥2	¥3	Choose nothing
Frequency of bonus payments	Paid every 2 weeks	Paid every 4 weeks	
Academic awards	Bonus points for physical education test scores	Bonus points for comprehensive test scores	
Frequency of exercise	5 times a week	1 time a week	
Exercise time	20 min each time	60 min each time	
Conditions for receiving the award	Complete the exercise program on a regular basis and upload it to the online platform	Register for the exercise incentive program	
	□	□	□

### 2.4. Data collection

This investigation employed a multi-stage sampling approach. Initially, 10 university students hailing from distinct schools in China’s eastern, central, and western regions were meticulously selected as enumerators, factoring in their geographical location and the economic development of the respective regions (amounting to a total of 30 enumerators). These adept enumerators were entrusted with the task of administering the questionnaires, with each one accountable for collecting 40–60 questionnaires. Prior to the survey, all enumerators underwent comprehensive and standardized training. The inclusion criteria comprised the following: (i) ordinary full-time undergraduates, encompassing both four-year and five-year programs, but excluding specialists; (ii) current residential students, excluding day students; (iii) individuals capable of participating in regular sports activities; and (iv) those proficient in independently completing the Chinese electronic questionnaire. Notably, no personally identifiable information was gathered in the questionnaire. In order to commence answering the questions and complete the questionnaire, respondents were required to select the “agree to participate in the survey” option, thereby signifying their voluntary engagement in the study. They were duly informed of the safeguarding of their privacy by law. Data collection for the survey spanned from 25 February to 25 March 2022.

### 2.5. Statistical analysis

Data analyses were conducted using lighthouse studio version 9.13.2 and SPSS (Statistical Package for the Social Sciences) version 25.0. The results of the descriptive analysis are presented as numbers of percentage stages regarding the participants’ general characteristics. In this study’s analysis, we employed a conditional logit model (CLM) (34, 35). CLM assist in assessing the influence of various attributes and levels on college students’ engagement in physical activity incentive programs and can gauge the relative significance of these attributes to college students. In this model, respondents’ choices served as the dependent variable, while the attributes investigated in the study were treated as the independent variables. The numerical representation of this model can be expressed as follows:


$$\begin{array}{l} U_{ijs}=\beta_{1}\mathrm{bonus}\;\left(2\right)+\beta_{2}\mathrm{bonus}\;\left(3\right)+\beta_{3}\mathrm{bonus}\;\left(4\right)\\ +\beta_{4}\mathrm{frequency of payments}\;\left(3\;\mathrm{weeks}\right)\\ +\beta_{5}\mathrm{frequency of payments}\;\left(2\;\mathrm{weeks}\right)\\ +\beta_{6}\mathrm{frequency of payments}\;\left(1\;\mathrm{week}\right)\\ +\beta_{7}\mathrm{academic awards}\;\left(\mathrm{physical education test scores}\right)\\ +\beta_{8}\mathrm{academic awards}\;\left(\mathrm{comprehensive test scores}\right)\\ +\beta_{9}\mathrm{frequency of exercise}\;\left(3\;\mathrm{times}\right)\\ +\beta_{10}\mathrm{frequency of exercise}\;\left(1\;\mathrm{time}\right)\\ +\beta_{11}\mathrm{exercise time}\;\left(40\;\min\right)\\ +\beta_{12}\mathrm{exercise time}\;\left(20\;\min\right)\\ +\beta_{13}\mathrm{Award conditions}\;\left(\mathrm{Completion of the incentive program}\right)\\ +\beta_{14}\mathrm{Award conditions}\;\left(\mathrm{Registered Incentive Program}\right)+\varepsilon_{ijs} \end{array}$$

Where *U_ijs_* is the utility for individual I for scenario j (*j* = 1, 2) in the choice set s (*s* = 1, 2, 3). *β* are a fixed vector of parameters for each attribute level.

We determined preference heterogeneity across classes, including *p*-value, Odds ratios (OR), and 95%CI, by digitally encoding features and levels. OR are metrics frequently employed in DCE to enhance comprehension of the CLM. The choice of the reference level for each characteristic serves as the foundation for the calculation of OR and 95% CI. Statistics on the respondents’ preference weights for each characteristic and level may be deduced from the CLM. Its sign—whether positive or negative—indicates the respondents’ preference.

We also represented the bonus amount as a continuous variable to compute respondents’ Willingness to Accept (WTA),as shown in the following formula. Amid the formula, *β*_X_ stands for nonprice attributes, and *β*_Price_ stands for price attributes.

WTA(X) = $\frac{\beta_{X}}{\beta_{\mathrm{Price}\text{.}}}$

When assessing a person’s WTP, we can determine how much they are willing to give up to choose one attribute level over another. WTA analysis via DCE has been applied to various interventions in different markets, including smoking cessation incentive programs, medication adherence incentive programs, vaccine preferences, and more (36–38). We want to use the WTA as an indicator to understand better university students’ preferences for various attributes and levels of the exercise incentive program.

Finally, we also conducted a subgroup analysis using propensity score matching (PSM) to understand the preferences of university students for various attributes of physical activity motivation strategies across gender, residence status, body mass index, and physical activity level. PSM is a regression method to identify patients in treatment and control groups with similar underlying characteristics. This method is commonly used in studies of impact factors, policy decisions, or case studies (39, 40). PSM is primarily based on the Roy-Robin theory (41–43). We matched each group of respondents according to their demographic characteristics (e.g., age, academic performance, and cost of living).

### 2.6. Ethics

This study was reviewed and approved by the ethics committees of the Shaanxi Health Culture Research Center (JKWH-2022-03). All methods were performed by the relevant guidelines and regulations (Declaration of Helsinki). Informed consent was obtained from all participants.

## 3. Results

### 3.1. Participants’ general information

Table 3 shows the general characteristics of each responder. A total of 1,475 respondents filled out the official survey; 1,358 (92.07%) of them passed the validity test, while 117 failed the logical tests’ one-choice sets. We thoroughly went over each respondent’s response before excluding them. 706 (51.9%) of the respondents who passed the validity test were female, and 55.45% were between the ages of 23 and over. A total of 28.06% of respondents were senior students, and 21.50% of respondents were first-year students. There were 179 (13.18%) respondents whose parents worked in sports-related industries. There are 170 (27.25%) respondents living from ¥1,101 to ¥1,400. 1,068 (78.65%) respondents had myopia problems, and only 211 respondents (15.54%) were without vision problems. A total of 182 (13.40%) respondents were overweight or obese on the Body Mass Index. 1,095 respondents had a high or medium level of physical activity, and 263 respondents had a low level of physical activity. 564 (41.53%) respondents spent more than 4 h a day using their cell phones. There are 605 (44.55%) respondents who never consumed alcohol and 1,187 (87.41%) respondents who never smoked or smoked for less than 6 months.

**Table 3:** General characteristics of all respondents (*N* = 1,358).

Characteristics	*N*	Proportion (%)
Gender		
Male	652	48.01
Female	706	51.99
Age (years)		
≤22	605	44.55
≥23	753	55.45
Nature of residence		
Non-agricultural residence	773	56.92
Agricultural residence	585	43.08
Grade Level		
Freshman year	292	21.50
Sophomore year	348	25.63
Third Year	337	24.81
Senior year	381	28.06
Parents in sports-related industries		
Yes	179	13.18
No	1,179	86.82
Living expenses(¥)		
≤800	53	3.90
801–1,100	228	16.79
1,101–1,400	370	27.25
1,401–1700	305	22.46
1701–2000	222	16.35
>2000	180	13.25
Vision Health		
No vision problems	211	15.54
Myopia	1,068	78.65
Hyperopia	37	2.72
Amblyopia	42	3.09
Astigmatism	329	24.23
Others	19	1.40
Body mass index (BMI)		
Thin	222	16.35
Normal	954	70.25
Overweight	157	11.56
Obesity	25	1.84
Physical activity level		
High	168	12.37
Medium	927	68.26
low	263	19.37
Hours of mobile phone use per day		
<1 h	43	3.17
1 ~ 2 h	94	6.92
2 ~ 3 h	353	25.99
3 ~ 4 h	304	22.39
>4 h	564	41.53
Frequency of alcohol consumption in the last year		
Never	605	44.55
No more than once a month	492	36.23
2–4 times per month	210	15.46
2–3 times per week	36	2.65
More than 4 times per week	15	1.11
Smoked continuously or cumulatively for 6 months		
Yes	171	12.59
No	1,187	87.41

### 3.2. University students’ preferences for the exercise incentive program

**Figure 1:**
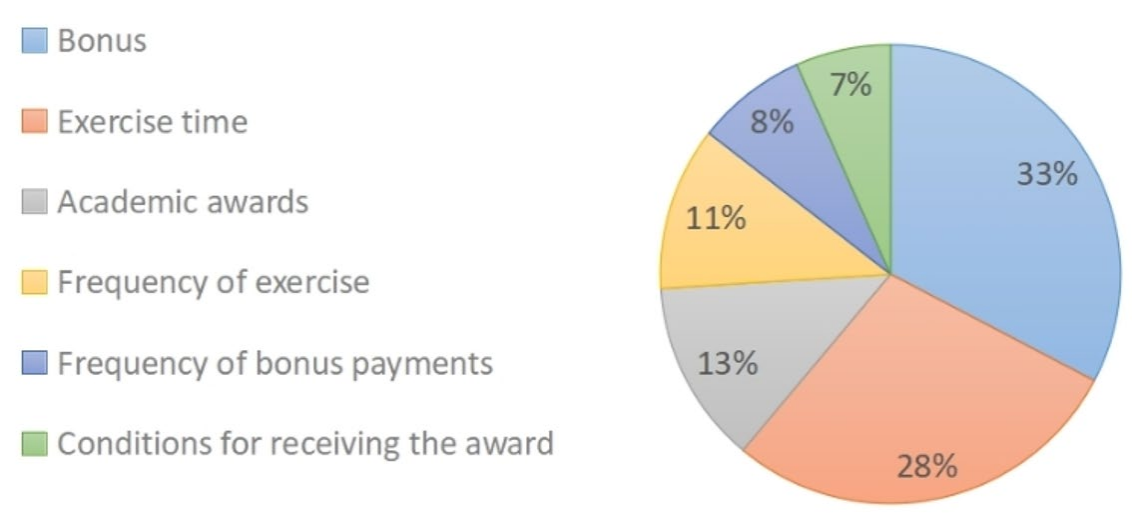
The importance of each attribute in all respondents.

Utilizing the CLM, we can ascertain the relative significance assigned by the respondents to each attribute of the exercise incentive program. Among the identified attributes, the “amount of the prize” emerged as the most pivotal, garnering a substantial importance score of 33%. Following closely, the attributes of “time to exercise” and “academic rewards” secured the second and third positions with significance scores of 28 and 13%, respectively. Comparatively, the attribute of “condition of receiving the reward” obtained the least prominence, bearing an importance score of 7% (Figure 1).

The results of the conditional logit model are shown in Table 4. It can be seen from the table that the utility of other attributes is statistically significant, except for “Frequency of bonus payments: paid every 3 weeks,” “Frequency of exercise: 3 times a week,” and” Conditions for receiving the award: Complete the exercise program regularly and upload it to the online platform. “As we expected, college students have different preferences for each level of attributes. Of all levels, “exercise time = 20 min each time” (*β* = 0.34, *p* < 0.001) is the most preferred of the respondents; the second is “amount of bonus = ¥4″ (*β* = 0.34, *p* < 0.001). It should not be ignored that the difference in utility between “paid every 1 week” (*β* = 0.07, *p* = 0.003) and “paid every 2 weeks” (*β* = 0.06, *p* = 0.006) is very little. For attribute academic awards, respondents preferred “bonus points for comprehensive test scores” (*β* = 0.17, *p* < 0.001) to other levels. Among attributes, frequency of exercise, and Conditions for receiving the award, respondents preferred “1 time a week” (*β* = 0.12, *p* < 0.001) and “Register for the exercise incentive program” (*β* = 0.06, *p* = 0.001).

**Table 4:** General results of the conditional logit model.

Attributes	*β*	SE	*t*	*p*	*OR*	95%CI	
Amount of bonus							
¥1^*^	−0.38	0.02	−16.16	<0.001	REF	REF	REF
¥2	−0.07	0.02	−3.18	0.002	1.36	1.30	1.42
¥3	0.11	0.02	5.06	<0.001	1.63	1.56	1.70
¥4	0.34	0.02	14.98	<0.001	2.04	1.95	2.13
Frequency of bonus payments							
paid every 4 weeks^*^	−0.10	0.02	−4.61	<0.001	REF	REF	REF
paid every 3 weeks	−0.02	0.02	−1.05	0.292	1.08	1.03	1.13
paid every 2 weeks	0.06	0.02	2.76	0.006	1.18	1.13	1.23
paid every 1 week	0.07	0.02	2.93	0.003	1.19	1.13	1.24
Academic awards							
bonus points for moral education credits^*^	−0.11	0.02	−6.09	<0.001	REF	REF	REF
bonus points for physical education test scores	−0.06	0.02	−3.38	0.001	1.05	1.01	1.09
bonus points for comprehensive test scores	0.17	0.02	9.53	<0.001	1.33	1.28	1.37
Frequency of exercise							
5 times a week^*^	−0.13	0.02	−7.09	<0.001	REF	REF	REF
3 times a week	0.01	0.02	0.29	0.769	1.14	1.10	1.18
1 time a week	0.12	0.02	6.89	<0.001	1.29	1.24	1.33
Exercise time							
60 min each time^*^	−0.28	0.02	−15.06	<0.001	REF	REF	REF
40 min each time	−0.07	0.02	−3.69	<0.001	1.23	1.19	1.28
20 min each time	0.34	0.02	19.08	<0.001	1.85	1.79	1.92
Conditions for receiving the award							
Pass the physical fitness test^*^	−0.09	0.02	−4.78	<0.001	REF	REF	REF
Complete the exercise program on a regular basis and upload it to the online platform	0.03	0.02	1.46	0.145	1.12	1.08	1.16
Register for the exercise incentive program	0.06	0.02	3.37	0.001	1.16	1.12	1.20

* REF, reference level.

In our calculation, we found that the odds ratio of some attributes’ levels is greater than 1, compared with the reference level, and the lower limit of 95% CI is also greater than 1. It means that college students are willing to choose other more advantageous levels than the reference level. Take the example of the attribute frequency of exercise. When using level “5 times a week” as a reference, the odds ratio for levels “3 times a week” and “1 time a week” are 1.14 (95%CI = 1.10 ~ 1.18) and 1.29 (95%CI = 1.24 ~ 1.33). As the attribute’ frequency of exercise’ decreases, the OR increases, meaning that university students prefer a lower exercise frequency in their choice of exercise incentive program. Therefore, university students prefer exercise incentive schemes that are less frequent and shorter, have more bonuses and payout bonuses more frequently, can increase total test scores and require only registration to receive awards.

### 3.3. Willingness to accept

The findings from the Willingness to Accept (WTA) estimation shed light on the comparison of college students’ preferences for the exercise incentive program concerning monetary aspects (Table 5). Based on the WTA results, it was observed that college students display a willingness to accept the highest cost to receive academic awards for total test scores (¥1.16). The second most significant aspects are engaging in each exercise session lasting 20 min (¥1.01) and exercising once a week (¥1.01). Regarding the frequency of bonus payments, there was marginal disparity in WTA between college students receiving a bonus every week (¥0.58) or every 2 weeks (¥0.60). Among the conditions for receiving rewards, their preference is to register for the exercise incentive program (¥0.48).

**Table 5:** Willingness to accept (WTA) for each attribute (*N* = 1,358).

Attributes and levels	WTA (¥)
Frequency of bonus payments	
paid every 4 weeks^*^	REF
paid every 3 weeks	0.28
paid every 2 weeks	0.60
paid every 1 week	0.58
Academic awards	
bonus points for moral education credits^*^	REF
bonus points for physical education test scores	0.21
bonus points for comprehensive test scores	1.16
Frequency of exercise	
5 times a week^*^	REF
3 times a week	0.60
1 time a week	1.01
Exercise time	
60 min each time^*^	REF
40 min each time	0.60
20 min each time	1.01
Conditions for receiving the award	
Pass the physical fitness test^*^	REF
Complete the exercise program on a regular basis and upload it to the online platform	0.38
Register for the exercise incentive program	0.48

* REF, reference level.

### 3.4. Differences in university students’ preferences for exercise incentive programs by different characteristics

Utilizing propensity score matching, we conducted an analysis of college students’ preferences for each attribute of the physical activity incentive program across gender, household nature, body mass index, and physical activity level. The pre-matching and post-matching comparisons across groups, along with the results from conditional logit models, have been presented in the Supplementary material.

In terms of attribute importance, both male and female participants considered “Amount of bonus” and “Exercise time” to be the two most crucial attributes. However, males attributed greater significance to “Amount of bonus” (43.38%), whereas females emphasized the importance of “Exercise time” (32.27%). The most preferred level for males was “Amount of bonus = ¥4” (*β* = 0.43, *p* < 0.001), while for females, it was “Exercise time = 20 min each time” (*β* = 0.47, *p* < 0.001).

Furthermore, for males, the attributes ranked in descending order of importance are “Academic rewards” (16.27%), “Frequency of bonus payments” (7.71%), “Frequency of exercise” (5.74%), and “Conditions for receiving the award” (3.78%). In contrast, for females, the order of importance is “Frequency of exercise” (14.85%), “Academic rewards” (9.70%), “Conditions for receiving the award” (9.35%), and “Frequency of bonus payments” (6.78%).

No significant difference in attribute importance was found between university students from non-agricultural and agricultural households. The ranking of importance for each attribute was as follows: “Amount of bonus,” “Exercise time,” “Academic rewards,” “Frequency of bonus payments,” “Frequency of exercise,” and “Conditions for receiving the award.”

We observed that university students with different body mass indexes displayed differing importance for the attributes of the incentive program. Thin and average university students considered “Amount of bonus” (36.79%) the most critical, followed by “Exercise time” (22.83%), “Academic rewards” (14.15%), “Frequency of exercise” (13.08%), “Frequency of bonus payments” (10.33%), and “Conditions for receiving the award” (2.81%). The most preferred level for them was “Amount of bonus = ¥4” (*β* = 0.46, *p* < 0.001).

On the other hand, overweight and obese university students valued “Exercise time” (28.03%) the most, followed by “Amount of bonus” (22.35%), “Academic rewards” (16.69%), “Frequency of bonus payments” (12.93%), “Conditions for receiving the award” (10.63%), and “Frequency of exercise” (9.37%). Their most preferred level was “Exercise time = 20 min each time” (*β* = 0.36, *p* < 0.001).

Additionally, we found that the physical activity level of university students influenced the relative importance of “Academic rewards” and “Frequency of exercise.” Those with a low level of physical activity considered “Academic rewards” (10.11%) to be more significant, while those with a medium or high level of physical activity valued “Frequency of exercise” (16.00%) more. For the remaining attributes, “Amount of bonus” and “Exercise time” remained the two most important attributes, with “Frequency of bonus payments” being the least important. The most preferred level for them was “Amount of bonus = ¥4.” For more detailed information, please refer to the Supplementary material.

## 4. Discussion

Through a comprehensive questionnaire survey and data analysis from 1,358 participants, we find that the attribute “bonus amount” emerges as the most critical and prioritized factor among the incentive measures, emphasizing the financial incentives in influencing college students. Among the six incentive attributes, attributes such as “single exercise duration” and “academic incentives” secure the second and third positions. Furthermore, college students prefer regular financial rewards for their accuracy, effectiveness, and dire financial needs. Additionally, academic rewards hold paramount importance for college students. Our study concludes that college students prefer immediate and tangible rewards.

Considering that college students necessitate stable financial incomes, they display heightened sensitivity to financial rewards, prompting a preference for regular bonuses. As a result, institutions can allocate a portion of their financial resources to incentive college students to participate in physical exercise. Simultaneously, initiating recurring sports competitions with associated rewards can effectively promote physical activities on campus. Furthermore, emphasizing the significance of physical health as the foundation for all activities, particularly for graduates embarking on their professional journey, it can foster the development of exercise habits before entering the workforce.

In tandem with the expansion of higher education, the competition among college students has become increasingly intense. Consequently, they attach great importance to academic performance, viewing all activities in the context of their academic achievements, which profoundly impact their future development. In the survey, respondents show favor the measure of “extra score points in a comprehensive test.” As a response, schools can appropriately enhance the proportion of physical education scores in the overall assessment of college students, thereby elevating its importance and motivating students to accord more attention to physical exercise. Moreover, various studies corroborate that physical exercise not only bears no detrimental effects on academic performance but also enhances cognitive abilities and academic achievements to a certain extent (44).

Moreover, our study indicates college students’ preference for short-term physical exercise. While some studies recommend a 90-min minimum for physical exercise to avoid harm to the body (45), particularly for individuals with irregular exercise habits, other studies have discovered that a 10-min set of joint exercises not only enhances physical fitness but also improves students’ attention and concentration (46). Additionally, studies involving children indicate that short-term aerobic exercise of varying intensity exerts selective positive effects on executive function (47). Therefore, even if college students engage in shorter and less frequent exercise sessions, it contributes to physical health, fitness, and academic performance. Furthermore, once an exercise habit is firmly established, it can significantly contribute to college students’ physical and psychological well-being over an extended period (48–50).

In parallel, we explore the differences in exercise incentive program preferences among university students based on various characteristics. Males exhibit a stronger preference for the attribute “Amount of bonus,” while females prioritize “Exercise time.” This difference may be attributed to gender disparities in personality and physiology (51). Moreover, the survey indicates a trend that females in our sample placed greater emphasis on the exercise process and duration of exercise, as these factors are often associated with weight management and overall well-being. Simultaneously, women often associate good health with a certain level of physical exercise. Additionally, university students with different body mass indexes display distinct preferences for incentive program attributes. For those with lower and average body mass indexes, the “Amount of bonus” ranks as the most critical attribute. Conversely, overweight and obese university students prioritize “Exercise time.” Average-weight students find physical exercise more manageable, allowing them to focus on completing exercise tasks effectively and obtaining rewards. In contrast, overweight or obese students recognize the challenges of exercise and are therefore more concerned about the time devoted to exercise. Consequently, schools should consider flexible adjustments based on students’ weight levels when devising strategies to motivate their participation in physical activities.

Finally, the demand for physical activity varies significantly among individuals with different exercise levels (52). Hence, accurately identifying the appropriate type and level of exercise suitable for the target audience is essential. Additionally, incorporating scientific, safe, and popular physical exercise methods that align with students’ interests can effectively promote physical exercise among college students (53). For instance, sports, dance, physical exercise, and fitness aerobics programs that align with college students’ psychological needs for physical attractiveness can enhance their interest in physical activities.

In this study, we investigated the preferences of college students concerning physical activity incentive programs. Furthermore, we applied propensity score matching to examine the variability in college students’ preferences for such programs across several factors like gender, family income level, and body mass index. The results of this analysis helped us to better comprehend the influence of demographic characteristics on their preferences for different features and levels of incentives. To ensure the quality and precision of the DCE, we collaborated with a team of experts specializing in sports, education, and methodology. After conducting an extensive literature review, we held interviews with these experts to identify the most relevant attributes and levels for the DCE.

However, it is essential to acknowledge certain limitations in this study. Firstly, given the vast population of college students in China, our sample data dose not fully encapsulate the entire college student population, thereby potentially leading to selection bias and impacting the generalizability of the findings. Despite the meticulous selection of attribute levels through literary reviews and expert advice, the hypothetical scenarios presented in the questionnaire may not perfectly mirror real-life situations. Moreover, this study focused on only six attributes of physical activity incentive programs, possibly overlooking other influential factors in such programs. Finally, the utilization of self-reported preferences from college students introduces subjectivity, which may affect the study’s degree of objectivity.

This study makes a substantial contribution to our comprehension of college students’ preferences for physical activity incentive programs. We employed propensity score matching to scrutinize these preferences, enhancing the statistical rigor of our analysis and allowing us to explore variations across factors such as gender and body mass index. To ensure the quality and precision of the Discrete Choice Experiment (DCE), we collaborated with a multidisciplinary team of experts specializing in sports, education, and research methodology. Following an extensive literature review, we conducted interviews with these experts to identify the most pertinent attributes and attribute levels for the DCE.

However, it is imperative to acknowledge several limitations in this study. Firstly, due to the vast population of college students in China, our sample data may not fully represent the entire college student population. This potential sampling bias could impact the generalizability of our findings. And we fail to provide a comprehensive analysis for more individual characteristics. Despite our meticulous selection of attribute levels through literature reviews and expert advice, the hypothetical scenarios presented in the questionnaire may not perfectly reflect real-life situations. Additionally, our study focused exclusively on six attributes of physical activity incentive programs, potentially overlooking other influential factors within these programs. Lastly, the reliance on self-reported preferences from college students introduces a degree of subjectivity, which may influence the objectivity of our study.

## 5. Conclusion

Based on the findings of a discrete choice experiment, this study presents college students’ preferences for physical activity incentive programs. The results reveal that prize amounts, exercise time, and academic rewards are the three most crucial attributes of the program. Students tend to prefer higher financial and academic rewards while minimizing their physical activity time. Furthermore, we observed variations in college students’ preferences for physical activity incentive programs based on individual characteristics such as gender, BMI, and physical activity level. These characteristics significantly influence students’ preferences for incentive programs. Therefore, it is recommended that educational institutions tailor physical activity incentive programs to meet the diverse needs of college students. This customization could involve designing varied reward structures that encompass financial and academic aspects and creating customized physical activity programs aligned with the unique characteristics of students of different genders and body weight levels. These strategies can enhance college students’ adherence to physical activity incentive programs, promoting a healthier and more active lifestyle among them.

## Data availability statement

The original contributions presented in the study are included in the article/Supplementary material, further inquiries can be directed to the corresponding authors.

## Ethics statement

The studies involving humans were approved by the ethics committees of the Shaanxi Institute of International Trade & Commerce. The studies were conducted in accordance with the local legislation and institutional requirements. Written informed consent for participation was not required from the participants or the participants’ legal guardians/next of kin in accordance with the national legislation and institutional requirements.

## Author contributions

JingbZ: Conceptualization, Data curation, Formal analysis, Investigation, Methodology, Project administration, Visualization, Writing – original draft. QL: Data curation, Formal analysis, Investigation, Methodology, Visualization, Writing – original draft, Conceptualization, Project administration. JingzZ: Formal analysis, Visualization, Writing – review & editing. XiaZ: Investigation, Writing – original draft. MJ: Writing – original draft. XH: Writing – original draft. DL: Investigation, Writing – review & editing. YY: Investigation, Writing – review & editing. XL: Investigation, Writing – review & editing. JC: Writing – review & editing. ZM: Writing – review & editing. XiyZ: Writing – review & editing. W-KM: Software, Writing – review & editing. T-hW: Writing – review & editing. GY: Conceptualization, Funding acquisition, Resources, Supervision, Writing – review & editing. YW: Conceptualization, Funding acquisition, Resources, Supervision, Writing – review & editing.
