# Supplementary material for: Chinese university students’ preferences for physical activity incentive programs: a discrete choice experiment
Source: Front Public Health. 2023 Nov 1;11:1281740. doi: 10.3389/fpubh.2023.1281740 (PMC10646335; doi:10.3389/fpubh.2023.1281740)
Supplement: Supplementary file 1 [file Table_1.DOCX]

Supplementary Material

**Results of PSM and conditional logit models based on gender characteristics**

There were 1358 respondents in this study, of whom 652 were male and 706 were female. We matched propensity scores using gender as the dependent variable(Male = 0, Female = 1) and age, nature of residence, grade, academic ranking, cost of living, myopia status and BMI as independent variables. There were 465 males and 465 females each after matching. Both groups before matching showed unevenness between groups in the re of household registration, grade, academic performance ranking, myopia, and BMI. The statistical characteristics before and after the PSM are shown in Appendix 1. The results based on the conditional logit show that the results of the overall sample do not differ significantly between males and females at the most preferred level for each attribute, with males most preferred at all levels for "Amount of bonus=¥4" (β=0.43, p<0.001), while the most preferred level for girls was "Exercise time=20 minutes each time" (β=0.47, p<0.001), as shown in Appendix 2. The most important attribute for boys was prize money (43.38%), while the most important attribute for girls was exercise time (32.27%), and the importance of each attribute is shown in Appendix 9.

**Appendix 1. Intergroup equilibrium before and after matching（gender as dependent variable）［n（%）］**

| Variable | Before matching | | | | After matching | | | |
| --- | --- | --- | --- | --- | --- | --- | --- | --- |
|  | **Male(n=652)** | **Female(n=706)** | χ^2^ | *P* | **Male(n=465)** | **Female(n=465)** | χ^2^ | *P* |
| **Age (years)** |  |  | 0.500 | 0.479 |  |  | 0.529 | 0.467 |
| ≤22 | 284（43.56） | 321（45.47） |  |  | 197(42.37) | 208(44.73) |  |  |
| ≥23 | 368（56.44） | 385（54.53） |  |  | 268(57.63) | 257(55.27) |  |  |
| **Nature of residence** |  |  | 14.682 | ＜0.001 |  |  | 0.531 | 0.466 |
| Non-agricultural residence | 406(62.27) | 367（52.98） |  |  | 271(58.28) | 260(55.91) |  |  |
| Agricultural residence | 246(37.73) | 339（48.02） |  |  | 194(41.72) | 205(44.09) |  |  |
| **Grade Level** |  |  | 133.526 | ＜0.001 |  |  | 0.000 | 1.000 |
| Freshman year | 93(14.26) | 199（28.19） |  |  | 88(18.92) | 88(18.92) |  |  |
| Sophomore year | 225(34.51) | 123（17.42） |  |  | 123(26.45) | 123(26.45) |  |  |
| Third Year | 205(31.44) | 132（18.70） |  |  | 125(26.88) | 125(26.88) |  |  |
| Senior year | 129(19.79) | 252（35.69） |  |  | 129(27.74) | 129(27.74) |  |  |
| **Academic Performance Ranking** |  |  | 9.110 | 0.028 |  |  | 6.291 | 0.098 |
| ≤10% | 130（19.94） | 123（17.42） |  |  | 95(20.43) | 84(18.06) |  |  |
| 11-30% | 183（28.07） | 242（34.28） |  |  | 137(29.46) | 166(35.70) |  |  |
| 31-50% | 177（27.15） | 200（28.33） |  |  | 125(26.88) | 130(27.96) |  |  |
| ＞50% | 162（24.85） | 141（19.97） |  |  | 108(23.23) | 85(18.28) |  |  |
| **Living expenses（¥）** |  |  | 7.280 | 0.063 |  |  | 6.897 | 0.075 |
| ≤1100 | 118（18.10） | 163（23.09） |  |  | 80(17.20) | 107(23.01) |  |  |
| 1101-1400 | 179（27.45） | 191（27.05） |  |  | 140(30.11) | 137(29.46) |  |  |
| 1401-1700 | 162（24.85） | 143（20.25） |  |  | 118(25.38) | 93(20.00) |  |  |
| ≥1701 | 193（29.60） | 209（29.60） |  |  | 127(27.31) | 128(27.53) |  |  |
| **Myopia** |  |  | 23.745 | ＜0.001 |  |  | 17.886 | ＜0.001 |
| Yes | 476（73.00） | 592（16.15） |  |  | 344(73.98) | 396(85.16) |  |  |
| No | 176（27.00） | 114（83.85） |  |  | 121(26.02) | 69(14.84) |  |  |
| **Body Mass Index（BMI）** |  |  | 6.201 | 0.013 |  |  | 2.35 | 0.125 |
| Thin and Normal | 549（84.20） | 627（88.81） |  |  | 394(84.73) | 410(88.17) |  |  |
| Overweight and Obesity | 103（15.80） | 79（11.19） |  |  | 71(15.27) | 55(11.83) |  |  |

**Appendix 2. Results of a conditional logit model for gender-specific university students.**

| **Attributes** | **Male(n=465)** | | | | | **Female(n=465)** | | | | |
| --- | --- | --- | --- | --- | --- | --- | --- | --- | --- | --- |
|  | ***β*** | ***P*** | ***OR*** | **95%CI** | | ***β*** | ***P*** | ***OR*** | **95%CI** | |
| **Amount of bonus** |  |  |  |  | |  |  |  |  |  |
| ¥1* | -0.43 | ＜0.001 | REF | REF | | -0.43 | ＜0.001 | REF | REF | |
| ¥2 | -0.12 | 0.002 | 1.36 | 1.27 | 1.47 | -0.03 | 0.392 | 1.48 | 1.37 | 1.60 |
| ¥3 | 0.12 | 0.002 | 1.73 | 1.61 | 1.86 | 0.14 | 0.001 | 1.76 | 1.63 | 1.90 |
| ¥4 | 0.43 | ＜0.001 | 2.35 | 2.18 | 2.53 | 0.33 | ＜0.001 | 2.12 | 1.97 | 2.29 |
| **Frequency of bonus payments** |  |  |  |  |  |  |  |  |  |  |
| paid every 1 week* | 0.06 | 0.115 | REF | REF | | 0.07 | 0.100 | REF | REF | |
| paid every 2 week | 0.06 | 0.105 | 1.00 | 0.93 | 1.08 | 0.04 | 0.312 | 0.98 | 0.90 | 1.05 |
| paid every 3 week | -0.03 | 0.412 | 0.91 | 0.85 | 0.98 | 0.02 | 0.629 | 0.96 | 0.88 | 1.03 |
| paid every 4 weeks | -0.09 | 0.018 | 0.86 | 0.80 | 0.93 | -0.12 | 0.002 | 0.83 | 0.77 | 0.90 |
| **Academic awards** |  |  |  |  |  |  |  |  |  |  |
| bonus points for moral education credits* | -0.13 | ＜0.001 | REF | REF | | -0.10 | 0.002 | REF | REF | |
| bonus points for physical education test scores | -0.05 | 0.126 | 1.09 | 1.03 | 1.16 | -0.07 | 0.025 | 1.03 | 0.97 | 1.09 |
| bonus points for comprehensive test scores | 0.18 | ＜0.001 | 1.38 | 1.30 | 1.46 | 0.17 | ＜0.001 | 1.31 | 1.23 | 1.39 |
| **Frequency of exercise** |  |  |  |  |  |  |  |  |  |  |
| 1 time a week* | 0.05 | 0.087 | REF | REF | | 0.20 | ＜0.001 | REF | REF | |
| 3 times a week | 0.01 | 0.753 | 0.96 | 0.90 | 1.02 | 0.01 | 0.757 | 0.83 | 0.78 | 0.88 |
| 5 times a week | -0.06 | 0.048 | 0.89 | 0.84 | 0.95 | -0.21 | ＜0.001 | 0.66 | 0.62 | 0.70 |
| **Exercise** **time** |  |  |  |  |  |  |  |  |  |  |
| 20 minutes each time* | 0.26 | ＜0.001 | REF | REF | | 0.47 | ＜0.001 | REF | REF | |
| 40 minutes each time | -0.07 | 0.018 | 0.71 | 0.67 | 0.76 | -0.04 | 0.190 | 0.60 | 0.56 | 0.64 |
| 60 minutes each time | -0.19 | ＜0.001 | 0.64 | 0.60 | 0.67 | -0.43 | ＜0.001 | 0.41 | 0.38 | 0.43 |
| **Conditions for receiving the award** |  |  |  |  |  |  |  |  |  |  |
| Register for the exercise incentive program* | 0.04 | 0.169 | REF | REF | | 0.07 | 0.019 | REF | REF | |
| Complete the exercise program on a regular basis and upload it to the online platform | -0.01 | 0.796 | 0.95 | 0.90 | 1.01 | 0.09 | 0.003 | 1.02 | 0.96 | 1.09 |
| Pass the physical fitness test | -0.03 | 0.274 | 0.93 | 0.88 | 0.99 | -0.17 | ＜0.001 | 0.79 | 0.74 | 0.84 |

**Results from PSM and conditional logit models based on the nature of residence**

There were 1358 respondents in this study, including 773 students in non-agricultural households and 585 students in agricultural households. We matched propensity scores using the nature of residence as the dependent variable (Non-agricultural residence = 0, Agricultural residence = 1) and gender, age, grade, academic ranking, cost of living, myopia status and BMI as the independent variables. There were 375 students in each of the two groups after matching. Both groups before matching showed unevenness between groups in gender and cost of living. The statistical characteristics before and after PSM are presented in Appendix 3. The results of the conditional logit showed that the two groups did not differ significantly from the results of the overall sample at the most preferred level for each attribute, with the most preferred level for both groups being "Amount of bonus=¥4 " (Non-agricultural residence: β=0.43, p<0.001; Agricultural residence: β=0.36, p<0.001), see Appendix 4. The most important attribute for both groups was the amount of bonus ( Non-agricultural residence: 35.45%; Agricultural residence: 35.94%), and the importance of each attribute is shown in Appendix 9.

**Appendix 3. Intergroup equilibrium before and after matching（nature of residence as dependent variable）［n（%）］**

| Variable | Before matching | | | | After matching | | | |
| --- | --- | --- | --- | --- | --- | --- | --- | --- |
|  | **Non-agricultural residence (n=773)** | **Agricultural residence (n=585)** | χ^2^ | *P* | **Non-agricultural residence (n=375)** | **Agricultural residence (n=375)** | χ^2^ | *P* |
| **Gender** |  |  | 14.628 | ＜0.001 |  |  | 0.000 | 1.000 |
| Male | 406（52.52） | 246（42.05） |  |  | 211(56.27) | 211(56.27) |  |  |
| Female | 367（47.48） | 339（57.95） |  |  | 164(43.73) | 164(43.73) |  |  |
| **Age (years)** |  |  | 3.671 | 0.055 |  |  | 0.347 | 0.556 |
| ≤22 | 327(42.30) | 278（47.52） |  |  | 160(42.67) | 168(44.80) |  |  |
| ≥23 | 446(57.70) | 307（52.48） |  |  | 215(57.33) | 207(55.20) |  |  |
| **Grade Level** |  |  | 7.010 | 0.135 |  |  | 0.000 | 1.000 |
| Freshman year | 161(20.83) | 131（22.39） |  |  | 49(13.07) | 49(13.07) |  |  |
| Sophomore year | 200(25.87) | 148（25.30） |  |  | 126(33.60) | 126(33.60) |  |  |
| Third Year | 210(27.17) | 127（21.71） |  |  | 114(30.40) | 114(30.40) |  |  |
| Senior year | 202(26.13) | 179（30.60） |  |  | 86(22.93) | 86(22.93) |  |  |
| **Academic Performance Ranking** |  |  | 0.225 | 0.973 |  |  | 3.547 | 0.315 |
| ≤10% | 144（18.63） | 109（18.63） |  |  | 63(16.80) | 76(20.27) |  |  |
| 11-30% | 244（31.57） | 181（30.94） |  |  | 127(33.87) | 105(28.00) |  |  |
| 31-50% | 216（27.94） | 161（27.52） |  |  | 108(28.80) | 115(30.67) |  |  |
| ＞50% | 169（21.86） | 134（22.91） |  |  | 77(20.53) | 79(21.07) |  |  |
| **Living expenses（¥）** |  |  | 129.537 | ＜0.001 |  |  | 4.731 | 0.193 |
| ≤1100 | 91（11.77） | 190（32.48） |  |  | 77(20.53) | 84(22.40) |  |  |
| 1101-1400 | 187（24.19） | 183（31.28） |  |  | 158(42.13) | 130(34.67) |  |  |
| 1401-1700 | 199（25.75） | 106（18.12） |  |  | 77(20.53) | 84(22.40) |  |  |
| ≥1701 | 296（38.29） | 106（18.12） |  |  | 63(16.80) | 77(20.53) |  |  |
| **Myopia** |  |  | 0.858 | 0.354 |  |  | 0.200 | 0.655 |
| Yes | 601（77.75） | 467（79.83） |  |  | 298(79.47) | 293(78.13) |  |  |
| No | 172（22.25） | 118（20.17） |  |  | 77(20.53) | 82(21.87) |  |  |
| **Body Mass Index（BMI）** |  |  | 0.755 | 0.385 |  |  | 0.402 | 0.526 |
| Thin and Normal | 664（85.90） | 512（87.52） |  |  | 320(85.33) | 326(86.93) |  |  |
| Overweight and Obesity | 109（14.10） | 73（12.48） |  |  | 55(14.67) | 49(13.07) |  |  |

**Appendix 4. Results of conditional logit models for university students in different resident locations.**

| **Attributes** | **Non-agricultural residence (n=375)** | | | | | **Agricultural residence (n=375)** | | | | |
| --- | --- | --- | --- | --- | --- | --- | --- | --- | --- | --- |
|  | ***β*** | ***P*** | ***OR*** | **95%CI** | | ***β*** | ***P*** | ***OR*** | **95%CI** | |
| **Amount of bonus** |  |  |  |  | |  |  |  |  |  |
| ¥1* | -0.48 | ＜0.001 | REF | REF | | -0.40 | ＜0.001 | REF | REF | |
| ¥2 | -0.08 | 0.056 | 1.49 | 1.37 | 1.63 | -0.08 | 0.062 | 1.37 | 1.26 | 1.49 |
| ¥3 | 0.14 | 0.002 | 1.86 | 1.71 | 2.02 | 0.12 | 0.005 | 1.68 | 1.54 | 1.82 |
| ¥4 | 0.43 | ＜0.001 | 2.50 | 2.29 | 2.72 | 0.36 | ＜0.001 | 2.12 | 1.95 | 2.31 |
| **Frequency of bonus payments** |  |  |  |  |  |  |  |  |  |  |
| paid every 1 week* | 0.03 | 0.494 | REF | REF | | 0.07 | 0.119 | REF | REF | |
| paid every 2 week | 0.11 | 0.015 | 1.08 | 0.99 | 1.18 | 0.08 | 0.061 | 1.01 | 0.93 | 1.10 |
| paid every 3 week | -0.02 | 0.674 | 0.95 | 0.88 | 1.04 | 0.01 | 0.742 | 0.95 | 0.87 | 1.03 |
| paid every 4 weeks | -0.12 | 0.007 | 0.86 | 0.79 | 0.94 | -0.16 | ＜0.001 | 0.80 | 0.73 | 0.87 |
| **Academic awards** |  |  |  |  |  |  |  |  |  |  |
| bonus points for moral education credits* | -0.10 | 0.003 | REF | REF | | -0.15 | ＜0.001 | REF | REF | |
| bonus points for physical education test scores | -0.08 | 0.021 | 1.02 | 0.96 | 1.10 | 0.01 | 0.890 | 1.17 | 1.09 | 1.25 |
| bonus points for comprehensive test scores | 0.19 | ＜0.001 | 1.33 | 1.25 | 1.43 | 0.15 | ＜0.001 | 1.35 | 1.26 | 1.44 |
| **Frequency of exercise** |  |  |  |  |  |  |  |  |  |  |
| 1 time a week* | 0.10 | 0.003 | REF | REF | | 0.09 | 0.007 | REF | REF | |
| 3 times a week | 0.02 | 0.629 | 0.92 | 0.86 | 0.98 | 0.02 | 0.506 | 0.93 | 0.87 | 1.00 |
| 5 times a week | -0.12 | 0.001 | 0.80 | 0.75 | 0.86 | -0.11 | 0.001 | 0.81 | 0.76 | 0.87 |
| **Exercise time** |  |  |  |  |  |  |  |  |  |  |
| 20 minutes each time* | 0.40 | ＜0.001 | REF | REF | | 0.29 | ＜0.001 | REF | REF | |
| 40 minutes each time | -0.05 | 0.140 | 0.63 | 0.59 | 0.68 | -0.02 | 0.632 | 0.74 | 0.69 | 0.79 |
| 60 minutes each time | -0.35 | ＜0.001 | 0.47 | 0.44 | 0.50 | -0.27 | ＜0.001 | 0.57 | 0.53 | 0.61 |
| **Conditions for receiving the award** |  |  |  |  |  |  |  |  |  |  |
| Register for the exercise incentive program* | 0.03 | 0.362 | REF | REF | | 0.05 | 0.118 | REF | REF | |
| Complete the exercise program on a regular basis and upload it to the online platform | 0.07 | 0.038 | 1.04 | 0.97 | 1.12 | -0.01 | 0.914 | 0.95 | 0.89 | 1.01 |
| Pass the physical fitness test | -0.10 | 0.003 | 0.87 | 0.82 | 0.94 | -0.05 | 0.153 | 0.90 | 0.85 | 0.97 |

**Results of PSM and conditional logit models based on BMI**

There were 1358 respondents in this study, of which 1176 students had a BMI of Thin and Normal and 182 students had a BMI of Overweight and Obesity. We matched propensity scores with BMI as the dependent variable (Thin and Normal=0, Overweight and Obesity=1) and gender, age, nature of household registration, grade, academic ranking, cost of living and myopia as the independent variables. The two groups were 120 each after matching. Both groups before matching showed unevenness between groups in gender and academic achievement ranking. The statistical characteristics before and after the PSM are presented in Appendix 5. The results of the conditional logit showed that the two groups did not differ significantly from the overall sample in terms of the most preferred level for each attribute, with those university students with a thin and normal BMI preferring "Amount of bonus=¥4" (β=0.46, p<0.001), while the most preferred level for overweight and obese students was "Exercise time=20 minutes each time" (β=0.36, p<0.001) The most important attribute for lean and normal students was bonus amount (36.79%), while the most important attribute for overweight and obese students was exercise time (28.03%), the importance of each attribute is shown in Appendix 9.

**Appendix 5. Intergroup equilibrium before and after matching（body mass index as dependent variable）［n（%）］**

| Variable | Before matching | | | | After matching | | | |
| --- | --- | --- | --- | --- | --- | --- | --- | --- |
|  | **Thin and Normal (n=1176)** | **Overweight and Obesity (n=182)** | χ^2^ | *P* | **Thin and Normal (n=120)** | **Overweight and Obesity (n=120)** | χ^2^ | *P* |
| **Gender** |  |  | 6.201 | 0.013 |  |  | 2.215 | 0.137 |
| Male | 549（46.68） | 103（56.59） |  |  | 93(77.50) | 102(85.00) |  |  |
| Female | 627（53.32） | 79（43.41） |  |  | 27(22.50) | 18(15.00) |  |  |
| **Age (years)** |  |  | 0.111 | 0.739 |  |  | 0.893 | 0.345 |
| ≤22 | 526（44.73） | 79（43.41） |  |  | 39(32.50) | 46(38.33) |  |  |
| ≥23 | 650（55.27） | 103（56.59） |  |  | 81(67.50) | 74(61.67) |  |  |
| **Nature of residence** |  |  | 0.755 | 0.385 |  |  | 0.000 | 1.000 |
| Non-agricultural residence | 664（56.46） | 109（59.89） |  |  | 68(56.67) | 68(56.67) |  |  |
| Agricultural residence | 512（43.54） | 73（40.11） |  |  | 52(43.33) | 52(43.33) |  |  |
| **Grade Level** |  |  | 3.317 | 0.506 |  |  | 0.024 | 0.999 |
| Freshman year | 255(21.68) | 37（20.33） |  |  | 18(15.00) | 18(15.00) |  |  |
| Sophomore year | 292(24.83) | 56（30.77） |  |  | 48(40.00) | 47(39.17) |  |  |
| Third Year | 295(25.09) | 42（23.08） |  |  | 38(31.67) | 39(32.50) |  |  |
| Senior year | 334(28.40) | 47（25.82） |  |  | 16(13.33) | 16(13.33) |  |  |
| **Academic Performance Ranking** |  |  | 11.111 | 0.011 |  |  | 7.291 | 0.063 |
| ≤10% | 229（19.47） | 24（13.19） |  |  | 21(17.50) | 16(13.33) |  |  |
| 11-30% | 735（31.89） | 50（27.47） |  |  | 42(35.00) | 34(28.33) |  |  |
| 31-50% | 325（27.64） | 52（28.57） |  |  | 36(30.00) | 31(25.83) |  |  |
| ＞50% | 247（21.00） | 56（30.77） |  |  | 21(17.50) | 39(32.50) |  |  |
| **Living expenses（¥）** |  |  | 0.819 | 0.845 |  |  | 5.907 | 0.116 |
| ≤1100 | 242（20.58） | 39（21.43） |  |  | 21(17.50) | 31(25.83) |  |  |
| 1101-1400 | 324（27.55） | 46（25.27） |  |  | 44(36.67) | 29(24.17) |  |  |
| 1401-1700 | 266（22.62） | 39（21.43） |  |  | 29(24.17) | 27(22.50) |  |  |
| ≥1701 | 344（29.25） | 58（31.87） |  |  | 26(21.67) | 33(27.50) |  |  |
| **Myopia** |  |  | 0.001 | 0.979 |  |  | 1.455 | 0.228 |
| Yes | 925（78.66） | 143（78.57） |  |  | 95(79.17) | 87(72.50) |  |  |
| No | 251（21.34） | 39（21.43） |  |  | 25(20.83) | 33(27.50) |  |  |

**Appendix 6. Results of a conditional logit model for university students with different body mass index.**

| **Attributes** | **BMI as thin and normal (n=120)** | | | | | **BMI as overweight and obesity (n=120)** | | | | |
| --- | --- | --- | --- | --- | --- | --- | --- | --- | --- | --- |
|  | ***β*** | ***P*** | ***OR*** | **95%CI** | | ***β*** | ***P*** | ***OR*** | **95%CI** | |
| **Amount of bonus** |  |  |  |  | |  |  |  |  |  |
| ¥1* | -0.43 | ＜0.001 | REF | REF | | -0.30 | ＜0.001 | REF | REF | |
| ¥2 | -0.15 | 0.059 | 1.33 | 1.14 | 1.55 | -0.02 | 0.781 | 1.32 | 1.14 | 1.53 |
| ¥3 | 0.12 | 0.109 | 1.74 | 1.51 | 2.02 | 0.18 | 0.021 | 1.61 | 1.39 | 1.87 |
| ¥4 | 0.46 | ＜0.001 | 2.45 | 2.11 | 2.85 | 0.15 | 0.057 | 1.56 | 1.35 | 1.81 |
| **Frequency of bonus payments** |  |  |  |  |  |  |  |  |  |  |
| paid every 1 week* | 0.12 | 0.120 | REF | REF | | -0.11 | 0.138 | REF | REF | |
| paid every 2 week | 0.02 | 0.839 | 0.90 | 0.78 | 1.04 | 0.08 | 0.314 | 1.21 | 1.04 | 1.41 |
| paid every 3 week | -0.01 | 0.989 | 0.89 | 0.77 | 1.03 | 0.16 | 0.040 | 1.31 | 1.13 | 1.52 |
| paid every 4 weeks | -0.13 | 0.082 | 0.78 | 0.67 | 0.90 | -0.12 | 0.115 | 1.00 | 0.86 | 1.15 |
| **Academic awards** |  |  |  |  |  |  |  |  |  |  |
| bonus points for moral education credits* | -0.17 | 0.006 | REF | REF | | -0.20 | 0.001 | REF | REF | |
| bonus points for physical education test scores | -0.01 | 0.945 | 1.18 | 1.05 | 1.33 | 0.05 | 0.423 | 1.29 | 1.14 | 1.45 |
| bonus points for comprehensive test scores | 0.17 | 0.006 | 1.41 | 1.25 | 1.59 | 0.16 | 0.012 | 1.43 | 1.27 | 1.61 |
| **Frequency of exercise** |  |  |  |  |  |  |  |  |  |  |
| 1 time a week* | 0.17 | 0.006 | REF | REF | | 0.02 | 0.724 | REF | REF | |
| 3 times a week | -0.03 | 0.649 | 0.82 | 0.73 | 0.92 | 0.09 | 0.133 | 1.07 | 0.95 | 1.20 |
| 5 times a week | -0.15 | 0.019 | 0.73 | 0.65 | 0.82 | -0.11 | 0.069 | 0.88 | 0.78 | 0.99 |
| **Exercise time** |  |  |  |  |  |  |  |  |  |  |
| 20 minutes each time* | 0.30 | ＜0.001 | REF | REF | | 0.36 | ＜0.001 | REF | REF | |
| 40 minutes each time | -0.04 | 0.567 | 0.72 | 0.64 | 0.81 | -0.11 | 0.066 | 0.63 | 0.56 | 0.71 |
| 60 minutes each time | -0.26 | ＜0.001 | 0.57 | 0.51 | 0.65 | -0.24 | ＜0.001 | 0.55 | 0.49 | 0.62 |
| **Conditions for receiving the award** |  |  |  |  |  |  |  |  |  |  |
| Register for the exercise incentive program* | -0.02 | 0.762 | REF | REF | | 0.11 | 0.074 | REF | REF | |
| Complete the exercise program on a regular basis and upload it to the online platform | -0.03 | 0.678 | 0.99 | 0.88 | 1.12 | 0.01 | 0.847 | 0.91 | 0.81 | 1.02 |
| Pass the physical fitness test | 0.04 | 0.480 | 1.06 | 0.94 | 1.20 | -0.12 | 0.052 | 0.80 | 0.71 | 0.90 |

**Results of PSM and conditional logit models based on physical activity levels**

There were 1358 respondents in this study, including 263 students with the low level of physical activity and 1095 students with medium and high levels of physical activity. We used physical activity level as the dependent variable (Low level of physical activity = 0, Medium and high levels of physical activity = 1) and gender, age, nature of household registration, grade level, academic ranking, cost of living, myopia status and BMI as independent variables for propensity score matching. The two groups were 164 each after matching. Both groups before matching showed unevenness between groups in gender, grade, academic achievement ranking, myopia, and BMI. The statistical characteristics before and after the PSM are presented in Appendix 7. The results of the conditional logit showed that the two groups did not differ significantly from the overall sample in the most preferred level of each attribute. bonus=¥4" (β=0.43, p<0.001), while "Exercise time=20 minutes each time" (β=0.43, p<0.001).The most important attribute for both groups was the length of exercise (Low level of physical activity: 31.28%; Medium and high levels of physical activity: 30.89%), and the importance of each attribute is shown in Appendix 9.

**Appendix 7. Intergroup equilibrium before and after matching（physical activity level as dependent variable）［n（%）］**

| **Variable** | **Before matching** | | | | **After matching** | | | |
| --- | --- | --- | --- | --- | --- | --- | --- | --- |
|  | **Low level of physical activity（n=263）** | **Medium and high levels of physical activity（n=1095）** | **χ^2^** | ***P*** | **Low level of physical activity（n=164）** | **Medium and high levels of physical activity（n=164）** | **χ^2^** | ***P*** |
| **Gender** |  |  | 37.027 | ＜0.001 |  |  | 0.335 | 0.563 |
| Male | 82（31.18） | 570（52.05） |  |  | 60(36.59) | 55(33.54) |  |  |
| Female | 181（68.82） | 525（47.95） |  |  | 104(63.41) | 109(66.46) |  |  |
| **Age (years)** |  |  | 2.672 | 0.102 |  |  | 0.444 | 0.505 |
| ≤22 | 129（49.05） | 476（43.47） |  |  | 88(53.66) | 94(57.32) |  |  |
| ≥23 | 134（50.95） | 619（56.53） |  |  | 76(46.34) | 70(42.68) |  |  |
| **Nature of residence** |  |  | 0.864 | 0.352 |  |  | 0.309 | 0.578 |
| Non-agricultural residence | 143（54.37） | 530（57.53） |  |  | 89(54.27) | 94(57.32) |  |  |
| Agricultural residence | 120（45.63） | 465（42.47） |  |  | 75(45.73) | 70(42.68) |  |  |
| **Grade Level** |  |  | 15.453 | 0.004 |  |  | 0.024 | 0.999 |
| Freshman year | 52(19.77) | 240（21.92） |  |  | 0(0.00) | 0(0.00) |  |  |
| Sophomore year | 53(20.15) | 295（26.94） |  |  | 33(20.12) | 33(20.12) |  |  |
| Third Year | 60(22.81) | 277（25.30） |  |  | 66(40.24) | 66(40.24) |  |  |
| Senior year | 98(37.27) | 283（25.84） |  |  | 65(39.63) | 65(39.63) |  |  |
| **Academic Performance Ranking** |  |  | 7.961 | 0.047 |  |  | 0.000 | 1.000 |
| ≤10% | 37（14.07） | 216（19.73） |  |  | 29(17.68) | 29(17.68) |  |  |
| 11-30% | 75（28.52） | 350（31.96） |  |  | 58(35.37) | 58(35.37) |  |  |
| 31-50% | 84（31.33） | 293（26.76） |  |  | 51(31.10) | 51(31.10) |  |  |
| ＞50% | 67（25.48） | 236（21.55） |  |  | 26(15.85) | 26(15.85) |  |  |
| **Living expenses（¥）** |  |  | 1.572 | 0.666 |  |  | 2.485 | 0.478 |
| ≤1100 | 55（20.91） | 226（20.64） |  |  | 32(19.51) | 38(23.17) |  |  |
| 1101-1400 | 77（29.28） | 293（26.76） |  |  | 52(31.71) | 55(33.54) |  |  |
| 1401-1700 | 52（19.77） | 253（23.10） |  |  | 33(20.12) | 36(21.95) |  |  |
| ≥1701 | 79（30.04） | 323（29.50） |  |  | 47(28.66) | 35(21.34) |  |  |
| **Myopia** |  |  | 6.456 | 0.011 |  |  | 0.203 | 0.653 |
| Yes | 222（84.41） | 846（77.26） |  |  | 25(15.24) | 28(17.07) |  |  |
| No | 41（15.59） | 249（22.74） |  |  | 139(84.76) | 136(82.93) |  |  |
| **Body Mass Index（BMI）** |  |  | 7.685 | 0.006 |  |  | 0.697 | 0.404 |
| Thin and Normal | 214（81.37） | 962（87.85） |  |  | 141(85.98) | 146(89.02) |  |  |
| Overweight and Obesity | 49（18.63） | 133（12.15） |  |  | 23(14.02) | 18(10.98) |  |  |

**Appendix 8. Results of a conditional logit model for university students with different levels of physical activity.**

| **Attributes** | **Low level of physical activity**  **（n=164）** | | | | | **Medium and high levels of physical activity（n=164）** | | | | |
| --- | --- | --- | --- | --- | --- | --- | --- | --- | --- | --- |
|  | ***β*** | ***P*** | ***OR*** | **95%CI** | | ***β*** | ***P*** | ***OR*** | **95%CI** | |
| **Amount of bonus** |  |  |  |  | |  |  |  |  |  |
| ¥1* | -0.30 | ＜0.001 | REF | REF | | -0.42 | ＜0.001 | REF | REF | |
| ¥2 | -0.08 | 0.245 | 1.25 | 1.10 | 1.42 | -0.06 | 0.374 | 1.43 | 1.26 | 1.63 |
| ¥3 | -0.06 | 0.348 | 1.26 | 1.11 | 1.44 | 0.15 | 0.028 | 1.75 | 1.54 | 1.99 |
| ¥4 | 0.43 | ＜0.001 | 2.07 | 1.82 | 2.36 | 0.33 | ＜0.001 | 2.11 | 1.85 | 2.40 |
| **Frequency of bonus payments** |  |  |  |  |  |  |  |  |  |  |
| paid every 1 week* | 0.01 | 0.915 | REF | REF | | -0.04 | 0.527 | REF | REF | |
| paid every 2 week | 0.04 | 0.581 | 1.03 | 0.91 | 1.17 | 0.10 | 0.137 | 1.15 | 1.01 | 1.31 |
| paid every 3 week | 0.07 | 0.275 | 1.07 | 0.94 | 1.21 | -0.01 | 0.871 | 1.03 | 0.91 | 1.17 |
| paid every 4 weeks | -0.11 | 0.082 | 0.89 | 0.78 | 1.01 | -0.05 | 0.493 | 1.00 | 0.88 | 1.13 |
| **Academic awards** |  |  |  |  |  |  |  |  |  |  |
| bonus points for moral education credits* | -0.09 | 0.098 | REF | REF | | -0.11 | 0.047 | REF | REF | |
| bonus points for physical education test scores | -0.06 | 0.227 | 1.02 | 0.92 | 1.14 | 0.01 | 0.912 | 1.12 | 1.01 | 1.24 |
| bonus points for comprehensive test scores | 0.15 | 0.004 | 1.27 | 1.15 | 1.40 | 0.10 | 0.061 | 1.23 | 1.11 | 1.36 |
| **Frequency of exercise** |  |  |  |  |  |  |  |  |  |  |
| 1 time a week* | 0.09 | 0.075 | REF | REF | | 0.23 | ＜0.001 | REF | REF | |
| 3 times a week | 0.05 | 0.386 | 0.95 | 0.86 | 1.06 | -0.07 | 0.198 | 0.74 | 0.67 | 0.82 |
| 5 times a week | -0.14 | 0.009 | 0.79 | 0.72 | 0.88 | -0.16 | 0.003 | 0.68 | 0.61 | 0.75 |
| **Exercise time** |  |  |  |  |  |  |  |  |  |  |
| 20 minutes each time* | 0.41 | ＜0.001 | REF | REF | | 0.43 | ＜0.001 | REF | REF | |
| 40 minutes each time | -0.07 | 0.163 | 0.62 | 0.56 | 0.69 | -0.10 | 0.055 | 0.59 | 0.53 | 0.65 |
| 60 minutes each time | -0.33 | ＜0.001 | 0.48 | 0.43 | 0.53 | -0.33 | ＜0.001 | 0.47 | 0.42 | 0.52 |
| **Conditions for receiving the award** |  |  |  |  |  |  |  |  |  |  |
| Register for the exercise incentive program* | 0.07 | 0.186 | REF | REF | | -0.01 | 0.816 | REF | REF | |
| Complete the exercise program on a regular basis and upload it to the online platform | 0.08 | 0.110 | 1.02 | 0.92 | 1.13 | 0.11 | 0.029 | 1.13 | 1.03 | 1.25 |
| Pass the physical fitness test | -0.15 | 0.004 | 0.80 | 0.72 | 0.89 | -0.10 | 0.057 | 0.92 | 0.83 | 1.02 |


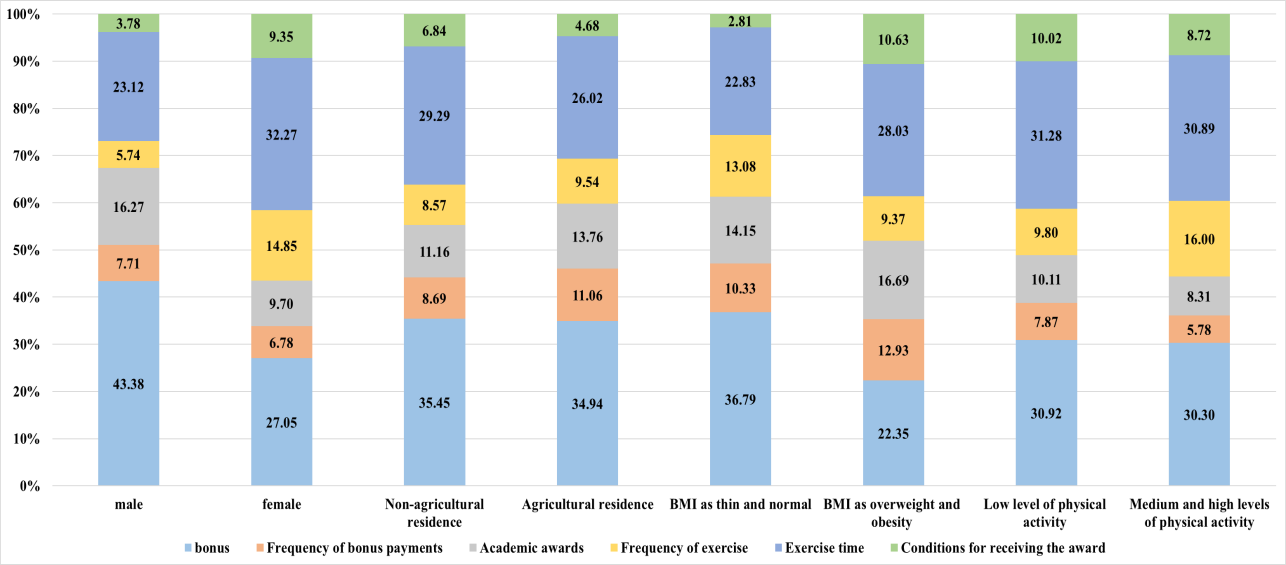


**Appendix 9．Importance of each attribute among different groups of respondents**
